# Ultrasound image-based nomogram combining clinical, radiomics, and deep transfer learning features for automatic classification of ovarian masses according to O-RADS

**DOI:** 10.3389/fonc.2024.1377489

**Published:** 2024-05-15

**Authors:** Lu Liu, Wenjun Cai, Hongyan Tian, Beibei Wu, Jing Zhang, Ting Wang, Yi Hao, Guanghui Yue

**Affiliations:** ^1^ Department of Ultrasound Medicine, South China Hospital, Medical School, Shenzhen University, Shenzhen, China; ^2^ Department of Ultrasound, Shenzhen University General Hospital, Medical School, Shenzhen University, Shenzhen, China; ^3^ National-Regional Key Technology Engineering Laboratory for Medical Ultrasound, Guangdong Key Laboratory for Biomedical Measurements and Ultrasound Imaging, School of Biomedical Engineering, Shenzhen University Medical School, Shenzhen University, Shenzhen, China

**Keywords:** ultrasound, machine learning, nomogram, ovarian cancer, O-RADS

## Abstract

**Background:**

Accurate and rapid discrimination between benign and malignant ovarian masses is crucial for optimal patient management. This study aimed to establish an ultrasound image-based nomogram combining clinical, radiomics, and deep transfer learning features to automatically classify the ovarian masses into low risk and intermediate-high risk of malignancy lesions according to the Ovarian- Adnexal Reporting and Data System (O-RADS).

**Methods:**

The ultrasound images of 1,080 patients with 1,080 ovarian masses were included. The training cohort consisting of 683 patients was collected at the South China Hospital of Shenzhen University, and the test cohort consisting of 397 patients was collected at the Shenzhen University General Hospital. The workflow included image segmentation, feature extraction, feature selection, and model construction.

**Results:**

The pre-trained Resnet-101 model achieved the best performance. Among the different mono-modal features and fusion feature models, nomogram achieved the highest level of diagnostic performance (AUC: 0.930, accuracy: 84.9%, sensitivity: 93.5%, specificity: 81.7%, PPV: 65.4%, NPV: 97.1%, precision: 65.4%). The diagnostic indices of the nomogram were higher than those of junior radiologists, and the diagnostic indices of junior radiologists significantly improved with the assistance of the model. The calibration curves showed good agreement between the prediction of nomogram and actual classification of ovarian masses. The decision curve analysis showed that the nomogram was clinically useful.

**Conclusion:**

This model exhibited a satisfactory diagnostic performance compared to junior radiologists. It has the potential to improve the level of expertise of junior radiologists and provide a fast and effective method for ovarian cancer screening.

## 1 Introduction

Ovarian masses comprise a remarkably diverse group of benign, borderline, and malignant lesions (1). The prognosis varies greatly depending on the histopathological type of lesions (2). Among these, ovarian cancer is the most lethal gynecological tumor (3) as more than 75% of patients with ovarian cancer are initially diagnosed at a late stage with a 5-year relative survival rate of only 29% (4). The treatment strategy for benign and malignant ovarian lesions is completely different. Conservative management or simple fertility-sparing resection is more appropriate for masses that are likely to be benign (5). Conversely, patients with suspicious malignant masses should be referred to a gynecologic oncologist and may require a more aggressive surgical approach (6). Therefore, the accurate preoperative classification of benign and malignant ovarian masses is crucial for optimal patient management.

Ultrasound scan is currently the first-line imaging modality for the screening of ovarian masses. With the extensive morphological characteristics displayed by ovarian masses, the interpretation of ultrasound images of these lesions is complex, and the accuracy of diagnosis is influenced by the experience and subjective judgment of radiologists. To establish standardized assessment procedures for adnexal masses, various evidence-based risk classification systems have been proposed to differentiate between benign and malignant adnexal masses (7–13). However, due to various reasons, their acceptance has been limited in clinical practice (14).

The Ovarian-Adnexal Reporting and Data System (O-RADS) risk stratification and management system for ultrasound, developed by the American College of Radiology (ACR) in 2020 and updated in 2022, classifies adnexal masses into six categories (O-RADS 0–5) representing the range of normal to high risk of malignancy. It provides a management recommendation for each risk category (14, 15). The clinical use of O-RADS is becoming more widespread, and its diagnostic performance has been validated for classifying benign and malignant lesions. An O-RADS 4 score has been identified as the optimal cutoff for malignancy characterization (16–18). Multiple retrospective studies have demonstrated that O-RADS has high sensitivity and specificity for classification (16–19). Although O-RADS has demonstrated excellent performance compared with other risk classification systems (20, 21), its apparent complexity and diverse presentation of ovarian lesions still pose a challenge for radiologists especially those in health resource-lacking regions with limited experience.

To provide rapid ultrasound image screening for ovarian cancer, address the shortage of medical resources, and assist less experienced radiologists in enhancing professional skills, intelligent diagnostic tools are needed to automatically classify the ovarian masses. Recently, with the development of artificial intelligence technology, the computer-assisted medical image analysis has enabled more accurate and reproducible evaluation for diseases, including ovarian diseases (22). In this study, we developed a nomogram combining radiomics, deep transfer learning (DTL), and clinical features to automatically categorize the ovarian masses into low risk of malignancy lesions (O-RADS 1–3) and intermediate-high risk of malignancy lesions (O-RADS 4–5) according to O-RADS. To the best of our knowledge, this specific subject has been rarely investigated until now.

## 2 Materials and methods

### 2.1 Ethical approval

This retrospective study was approved by the ethical committees of the South China Hospital of Shenzhen University (approval number: HNLS20230112101-A). As the study was conducted retrospectively, the requirement for patient informed consent was waived.

### 2.2 Patients and data acquisition

**Figure 1:**
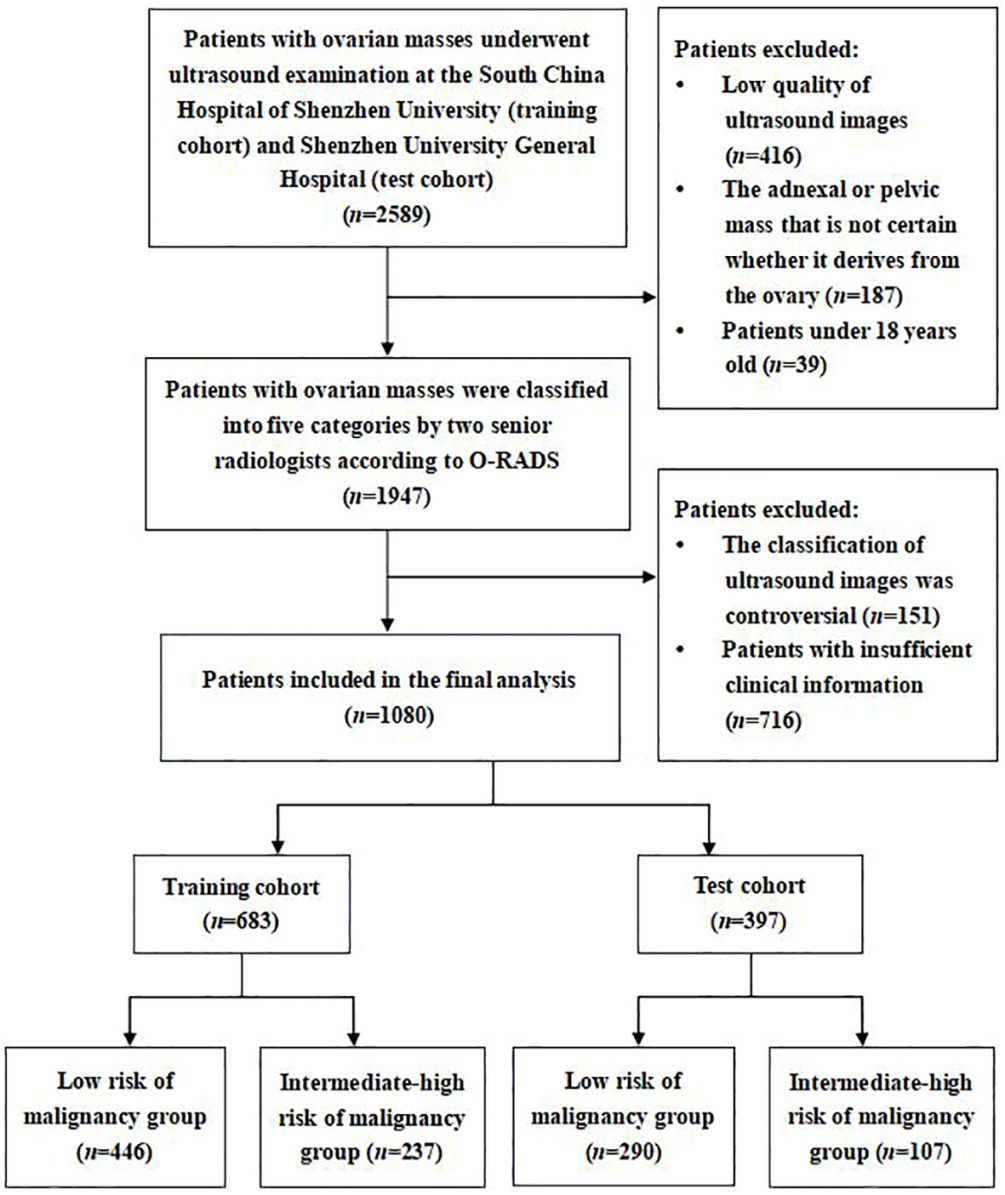
Flowchart of the study subjects’ screening based on inclusion and exclusion criteria.

Between July 2021 and December 2023, we retrospectively collected transvaginal or transrectal ultrasound images of patients with ovarian masses who underwent ultrasound examination at the South China Hospital of Shenzhen University as the training cohort. Meanwhile, the ultrasound images of ovarian masses collected from the Shenzhen University General Hospital were regarded as the test cohort. Both hospitals are general hospitals rather than reference oncology centers. According to the O-RADS ultrasound risk stratification and management system, two senior radiologists (W.C. and H.T.) with over 20 years of experience in gynecological ultrasonography classified these ultrasound images of ovarian masses into five categories (O-RADS 1–5). As O-RADS 4 has been shown to be an appropriate cutoff for malignancy (16, 18), ovarian mass classified as O-RADS 1–3 was considered as benign probable lesion with low risk of malignancy (<10%), while ovarian mass classified as O-RADS 4–5 was considered as malignant probable lesion with intermediate-high risk of malignancy (≥10%). Therefore, these ultrasound images were divided into the following two groups: a low risk of malignancy group (O-RADS 1–3) and an intermediate-high risk of malignancy group (O-RADS 4–5) based on the judgment of senior radiologists. The senior radiologists’ consistent classification of the images served as the diagnostic criterion for validating the diagnostic performance of the models and junior radiologists. If they disagreed on the classification of an ovarian mass, they would consult with the third senior radiologist (L.L.) for resolution (**Figure 1**).

The inclusion criteria were as follows: (1) Patients with ovarian masses who underwent transvaginal or transrectal sonography scan at the South China Hospital of Shenzhen University or Shenzhen University General Hospital. (2) Patients over 18 years old. (3) We only included one ovarian mass per patient. If there were more than one mass, the mass with the most complex morphology or the largest diameter was included. The exclusion criteria were as follows: (1) Adnexal or pelvic mass that is uncertain whether it derives from the ovary; (2) Ultrasound images with low quality that were unsuitable for further analysis; (3) The clinical information of patient was incomplete.

### 2.3 Clinical characteristics

The clinical characteristics of the patients with ovarian masses we collected included the age of patients, maximum diameter of the lesion, presenting symptoms, menopause status, and presence or absence of ascites. The symptoms included dysmenorrhea, dyspareunia, chronic pelvic pain, abdominal pain, and abdominal fullness. The frequency of symptoms was at least three times a month.

### 2.4 Ultrasound image acquisition

Ultrasound scans were conducted using different machines equipped with transvaginal probes, including GE Voluson E10, GE Logiq E9, Mindray DC-80, and Samsung HERA XW10. All transvaginal or transrectal ultrasound scans were performed by certified radiologists with more than 3 years of experience in gynecological ultrasonography. Typically, an ovarian mass may have multiple images, and the one with the maximum lesion diameter was selected. However, if a patient had more than one mass, the one with the most complex morphology or the largest diameter was chosen for analysis. After image quality control conducted by three radiologists (B.W., J.Z., and T.W.), images that met the inclusion criteria were extracted from the Picture Archiving and Communication Systems in JPEG format.

### 2.5 Image segmentation, feature extraction, and feature fusion

**Figure 2:**
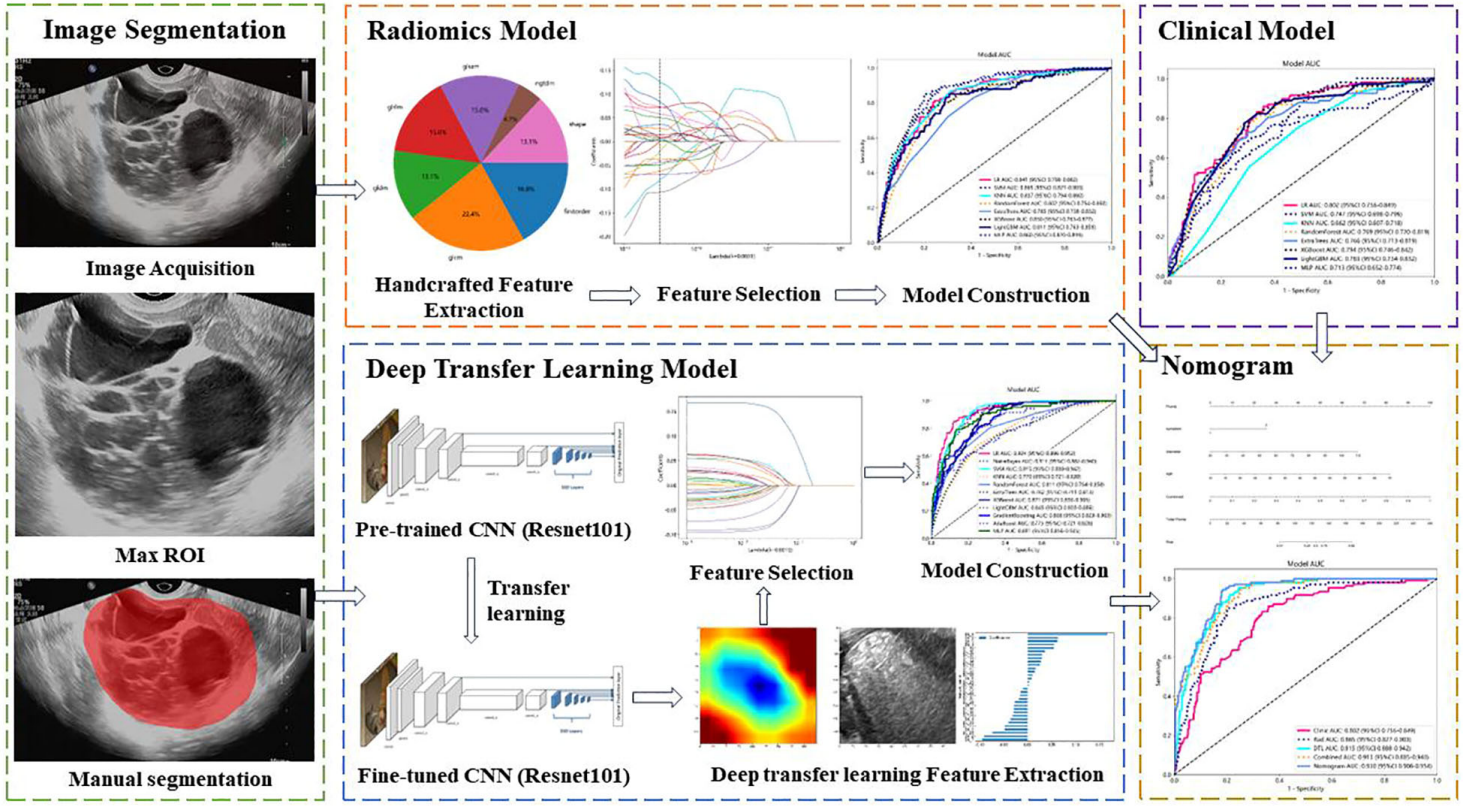
Workflow of ultrasound-based nomogram construction. ROI, regions of interest. CNN, convolutional neural network.

The workflow of the ultrasound-based deep learning radiomics nomogram analysis included image segmentation, feature extraction, feature selection, and model construction (**Figure 2**). The included ultrasound images were converted to the NII format. Two independent investigators (L.L. and W.C.) who were blinded to the classification results reviewed these images and used ITK-SNAP software (Version 3.8.0, USA) to manually segment the regions of interest (ROIs) of target lesions. The interclass correlation coefficient (ICC) was used to evaluate the intra-/inter-observer agreement and reproducibility of the feature extraction. An ICC value of ≥0.75 was considered indicative of a satisfactory agreement.

The radiomics features were handcrafted features extracted using the Pyradiomics analysis program, a web-based tool for radiomics analysis (http://pyradiomics.readthedocs.io). Filters were used to generate derived images. The extracted features can be categorized into geometry, intensity, and texture features. There are five types of texture features, including gray-level co-occurrence matrix (GLCM), gray-level dependence matrix (GLDM), gray-level size zone matrix (GLSZM), gray-level run length matrix (GLRLM), and neighboring gray tone difference matrix (NGTDM). Geometry features describe the shape characteristics of the lesions. Intensity features depict the first-order statistical distribution of the voxel intensities within the lesions. Texture features describe the patterns or the second- and high-order spatial distributions of the intensities.

Deep learning features refer to the features extracted using deep learning networks, which are manifested as learned weights from networks. The deep learning features were extracted from pre-trained convolutional neural networks (CNN) via transfer learning to overcome the overfitting problems that deep learning models usually suffer from due to insufficient training data. The parameters of several CNNs were trained, including Resnet-50, Resnet-101, Resnet-152, Densenet-121, Densenet-201, and Inception v3. Then, these pre-trained DTL networks were used to extract deep learning features, and the optimal model was selected. The image files were converted from JPEG to PNG format for further analysis. The penultimate layer output features, expressed as activation values, were extracted as deep learning features representing the high-level visual patterns from the images by the pre-trained CNN. The principal component analysis (PCA) was used to reduce the dimension of DTL features, improve the generalization ability of the model, and reduce the risk of overfitting.

The extracted radiomics features were used to establish the radiomics model, and the extracted DTL features were used to establish the DTL model. To improve the performance of classification for ovarian masses, we fused clinical features, radiomics features, and DTL features to obtain the optimal subset of fusion features. The fusion strategy of concatenation sum was used to fuse these different features. The fusion scheme included clinical features combined with radiomics features, radiomics features combined with DTL features, and clinical features combined with radiomics features and DTL features. First, we combined clinical features with radiomics features. Then, we combined radiomics features with DTL features. Finally, the clinical features, radiomics features, and DTL features were all combined to establish the nomogram.

### 2.6 Feature selection and model construction

All mono-modal and fusion features were standardized using the Z-score method, and the mean and variance of each feature was calculated. Each feature was then subtracted from the mean, divided by variance, and transformed into a standard normal distribution. To select the features most correlated with the classification outcome of ovarian masses, we used the *t*-test or Mann–Whitney *U*-test for feature screening. Only radiomic features with a p-value <0.05 were kept to select the features with significant differences between two groups. To delete the redundant features, we calculated the correlation between features using Spearman’s rank correlation coefficient to evaluate their multi-collinearity. We retained only one of the features with a correlation coefficient greater than 0.9 between any two features to delete those with high repeatability. Additionally, we performed a greedy recursive deletion strategy (the feature with the greatest redundancy in the current set is deleted each time) for feature filtering to retain the ability to accurately describe features to the greatest extent.

Subsequently, employing the scikit-learn package in Python (version 3.70), the least absolute shrinkage and selection operator (LASSO) regression model was used to select and reduce the number of features for model construction. Depending on the regulation weight λ, LASSO shrinks all regression coefficients toward zero and sets the coefficients of the irrelevant features exactly to zero. Using 10-fold cross-validation with minimum criteria, the optimal λ was determined, where the final value of λ resulted in the minimum cross-validation error. The most robust non-redundant retained features with non-zero coefficients were used for regression model fitting and combined into a radiomics signature. The retained features with non-zero coefficients were selected to establish the score using a LASSO logistic regression model. Finally, a score for each patient was obtained by a linear combination of the retained features weighed by their model coefficients. Similarly, based on the concatenation sum of radiomics features and DTL features, we also used LASSO regression model to select the fusion features.

After feature screening with LASSO, we employed the scikit-learn package in Python (version 3.70) to construct and assess the radiomics model, DTL model, clinical model, and fusion model. Using the final selected features, we input them into a variety of machine learning models, such as logistic regression (LR), support vector machine (SVM), k-nearest neighbor (KNN), random forest, XGBoost, LightGBM, multi-layer perception (MLP), NaiveBayes, and GradientBoosting to construct the models. Subsequently, we conducted fivefold cross-verification to determine the optimal model hyperparameters for model fitting and obtained the final signature that is most robust and non-redundant. Finally, a nomogram model fusing the clinical, radiomics, and DTL features was established for final interpretation and analysis. The nomogram was constructed using multivariate logistic regression to combine the scores of these features developed on the training cohort. A nomogram score was then calculated for each patient in both the training and test cohorts to predict the risk of malignancy with this score combining the clinical, radiomics, and DTL scores weighted by their respective coefficients.

The diagnostic efficacy of different models was comprehensively evaluated in test cohort, and receiver operating characteristic (ROC) curves were plotted to visually assess the diagnostic performance of these models. Additionally, various diagnostic indices were calculated, including area under the ROC curve (AUC), specificity, sensitivity, accuracy, positive predictive value (PPV), negative predictive value (NPV), and precision. The DeLong test was conducted to compare the AUCs of different models using MedCalc software (version 20.100). To compare the agreement between the prediction of the nomogram and the actual classification, calibration curves were drawn to evaluate the calibration efficiency of the nomogram, and Hosmer–Lemeshow analysis was used to assess the calibration ability of nomogram. Furthermore, decision curve analysis (DCA) was conducted to evaluate the clinical utility of the predictive models.

### 2.7 Evaluation and comparison with radiologists

Two senior radiologists (W.C. and H.T.) independently evaluated and classified all ultrasound images in the training cohort and test cohort according to O-RADS. Both of them were expert gynecological radiologists with over 20 years of clinical experience. Three junior radiologists (B.W., J.Z., and T.W.) with less than 5 years of experience in gynecological ultrasonography were assigned to independently classify the images in the test cohort. All selected ovarian masses were evaluated for the following ultrasound features: lesion category (unilocular or multilocular cyst with or without solid component), size (maximum diameters), external/outer contour (smooth or irregular), internal contents (hypoechoic/isoechoic/hyperechoic, calcification, and acoustic shadowing), internal content (anechoic fluid, hyperechoic components, scattered low-level echoes, and fluid level), septations (complete and incomplete), and solid or solid/appearing component (papillary projection or nodule). All radiologists were blinded to the prediction results of the models and original ultrasound reports. After a period of time, the junior radiologists were instructed to reevaluate and reclassify each image with the assistance of the nomogram. The junior radiologists may refer to the results from the nomogram to make a change in their classification especially when they were not sure of how to classify them.

### 2.8 Statistical analysis

Statistical package IBM SPSS (version 21.0) was used to compare the clinical characteristics of the patients between two groups. The continuous variables, such as patients’ age and maximum diameter of the lesions, were described as mean ± standard deviation and analyzed using *t*-test or Mann–Whitney *U*-test. The categorical variables, including presenting symptom, menopause status, and presence or absence of ascites, were described as frequencies and percentages and analyzed using Chi-square test. Statistical significance was defined as a two-sided p-value <0.05. The 95% confidence interval (CI) of AUC was calculated. Additionally, Python (version 3.70) was used to perform the ICCs, Spearman rank correlation test, Z score normalization, and LASSO regression analysis.

## 3 Results

### 3.1 Comparison of patients’ clinical characteristics

In this study, we included 1,080 patients with 1,080 ovarian masses. The training cohort consisted of 683 patients, 446 in the low risk of malignancy group and 237 in the intermediate-high risk of malignancy group based on the classification of senior radiologists according to the O-RADS. The test cohort consisted of 397 patients, 290 in the low risk of malignancy group and 107 in the intermediate-high risk of malignancy group. **Figure 1** illustrates the flowchart outlining the screening process of participants according to the inclusion and exclusion criteria. **Table 1** provides a comparison of the baseline clinical characteristics of the patients between the two groups.

**Table 1:** Baseline clinical characteristics of participants between two groups.

Clinical features	Training All (*n* = 683)	Training Low risk of malignancy group (*n* = 446)	Training Intermediate-high risk of malignancy group (*n* = 237)	p-Value	Test All (*n* =397)	Test Low risk of malignancy group (*n* = 290)	Test Intermediate-high risk of malignancy group (*n* = 107)	p-Value
Age (years)	34.71 ± 8.78	32.38 ± 7.76	39.11 ± 8.93	<0.001	35.58 ± 7.56	34.16 ± 7.30	39.44 ± 6.90	<0.001
Diameter (mm)	42.92 ± 12.70	40.45 ± 12.17	47.56 ± 12.41	<0.001	41.90 ± 11.22	40.23 ± 10.18	46.40 ± 12.64	<0.001
Symptom				<0.001				0.001
0	546 (79.94%)	319 (71.52%)	227(95.78%)		324 (81.61%)	225 (77.59%)	99 (92.52%)	
1	137 (20.06%)	127 (28.48%)	10 (4.22%)		73 (18.39%)	65 (22.41%)	8 (7.48%)	
Menopause				<0.001				<0.001
0	632 (92.53%)	428 (95.96%)	204 (86.08%)		378 (95.21%)	283 (97.59%)	95 (88.79%)	
1	51 (7.47%)	18 (4.04%)	33 (13.92%)		19 (4.79%)	7 (2.41%)	12 (11.21%)	
Ascites				0.042				0.029
0	675 (98.83%)	444 (99.55%)	231 (97.47%)		392 (98.74%)	289 (99.66%)	103 (96.26%)	
1	8 (1.17%)	2 (0.45%)	6 (2.53%)		5 (1.26%)	1 (0.34%)	4 (3.74%)	

Symptom, menopause, and ascites 0 mean the participants were asymptomatic, non-menopausal status, and absence of ascites, respectively. Symptom, menopause, and ascites 1 mean the participants were symptomatic, menopausal status, or presence of ascites.

Significant differences were observed in all clinical characteristics between the two groups in both the training cohort and test cohort (p < 0.05). In the training cohort, the mean age of the low risk of malignancy group and intermediate-high risk of malignancy group was 32.38 ± 7.76 and 39.11 ± 8.93 years, respectively (p < 0.001). The maximum diameter of lesion was 40.45 ± 12.17 and 47.56 ± 12.41 mm in the low risk of malignancy group and intermediate-high risk of malignancy group, respectively (p < 0.001). In the low risk of malignancy group, 28.48% of the patients had clinical symptoms, whereas 4.22% of the patients in the intermediate-high risk of malignancy group had clinical symptoms (p < 0.001). The proportion of postmenopausal patients was 4.04% and 13.92% in the low risk of malignancy group and intermediate-high risk of malignancy group, respectively (p < 0.001). The proportion of patients with ascites was 0.45% and 2.53% in the low risk of malignancy group and intermediate-high risk of malignancy group, respectively (p = 0.042).

In the test cohort, the mean age was 34.16 ± 7.30 and 39.44 ± 6.90 years in the low risk of malignancy group and intermediate-high risk of malignancy group, respectively (p < 0.001). The maximum diameter of lesion was 40.23 ± 10.18 mm in the low risk of malignancy group and 46.40 ± 12.64 mm in the intermediate-high risk of malignancy group (p < 0.001). In the low risk of malignancy group, 22.41% of the patients had clinical symptoms, whereas 7.48% of the patients in the intermediate-high risk of malignancy group had clinical symptoms (p = 0.001). The proportion of postmenopausal patients was 2.41% and 11.21% in the low risk of malignancy group and intermediate-high risk of malignancy group, respectively (p < 0.001). The proportion of patients with ascites was 0.34% and 3.74% in the low risk of malignancy group and intermediate-high risk of malignancy group, respectively (p = 0.029).

### 3.2 Feature extraction and selection

**Figure 3:**
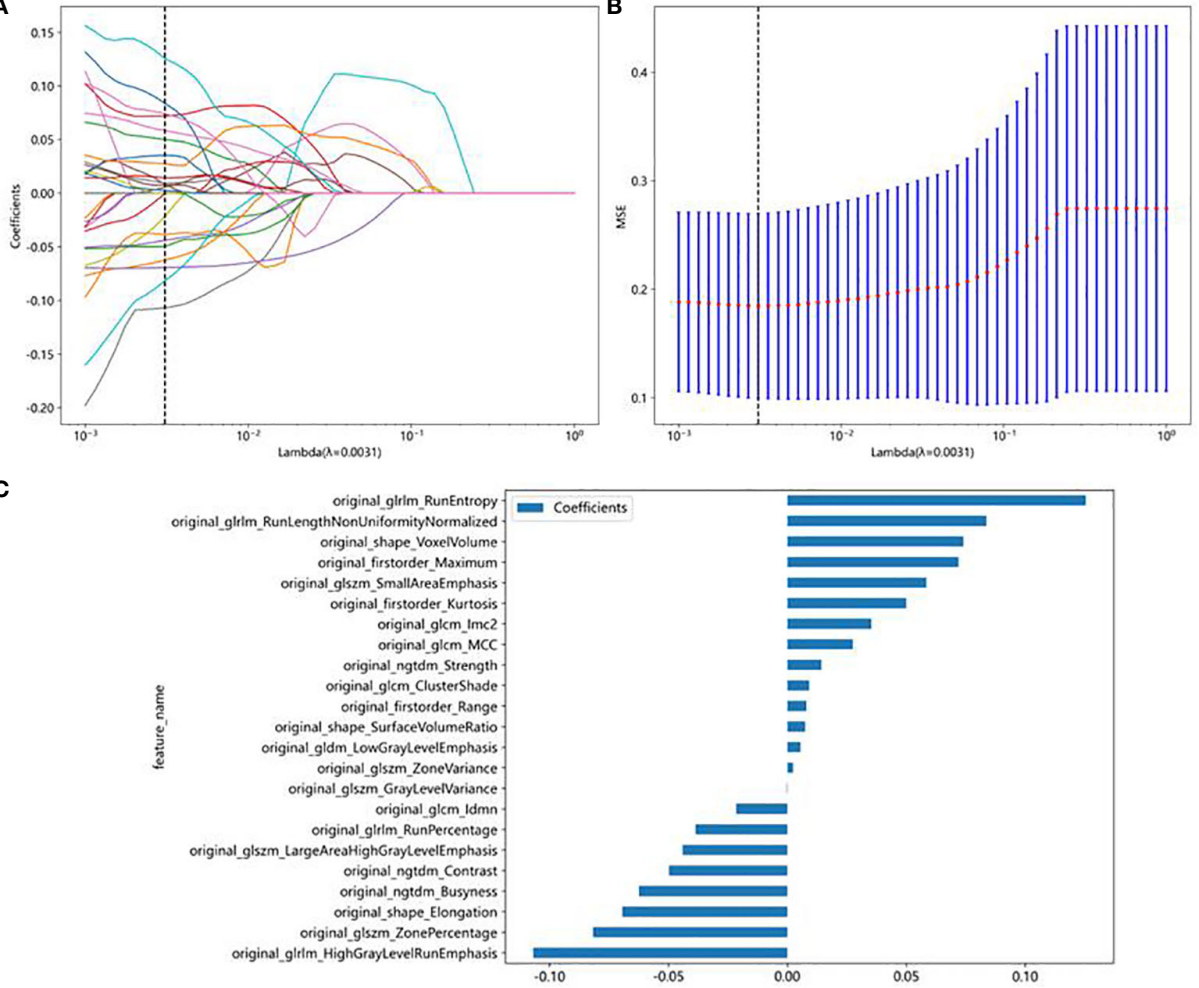
Radiomics feature selection using the least absolute shrinkage and selection operator (LASSO) logistic regression model in the test cohort. **(A)** Coefficients of 10-fold cross-validation based on LASSO algorithm. **(B)** MSE of 10-fold cross-validation based on LASSO algorithm. **(C)** Histogram depicting the values of coefficients in the final selected non-zero features.

**Figure 4:**
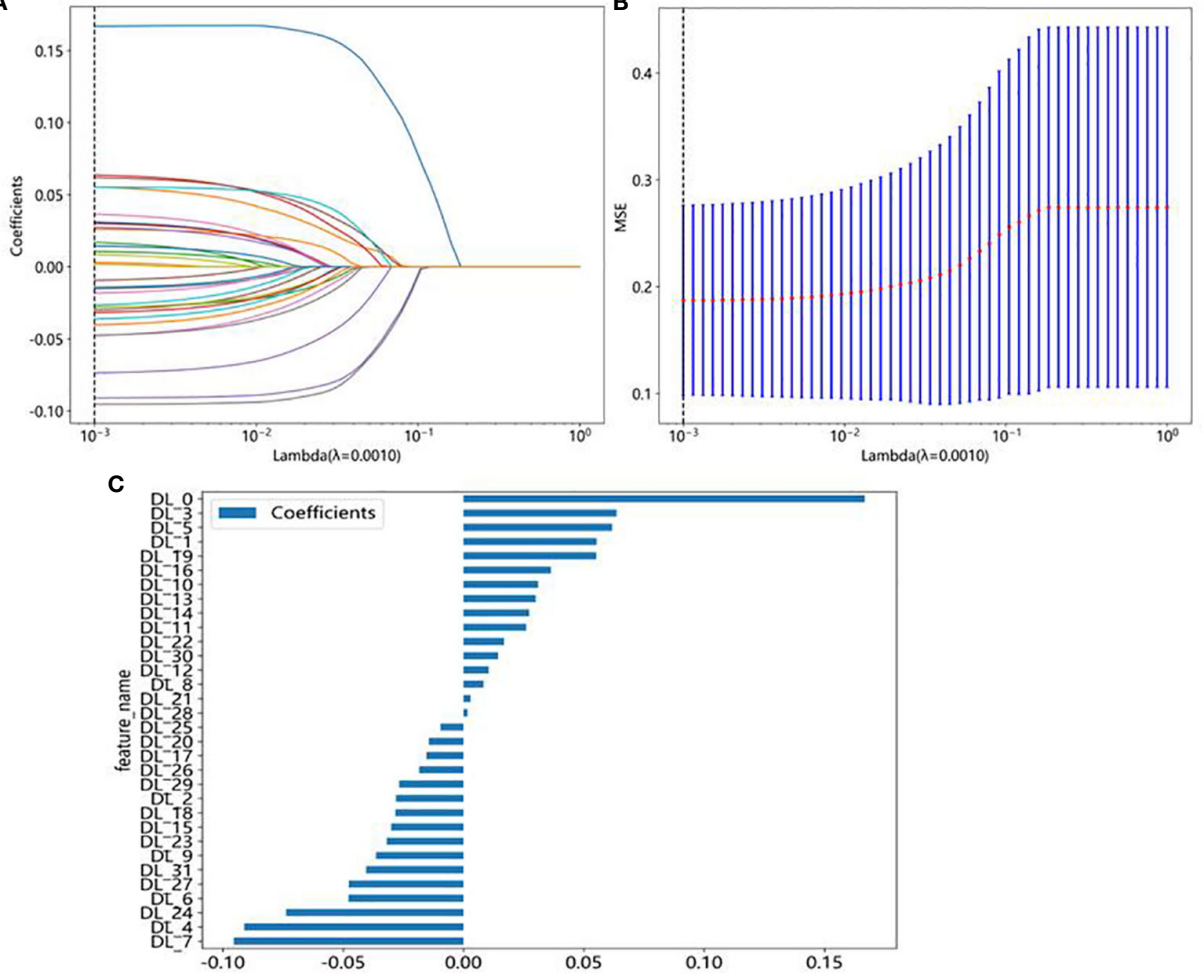
DTL feature selection using the least absolute shrinkage and selection operator (LASSO) logistic regression model in the test cohort. **(A)** Coefficients of 10-fold cross-validation based on LASSO algorithm. **(B)** MSE of 10-fold cross-validation based on LASSO algorithm. **(C)** Histogram depicting the values of coefficients in the final selected non-zero features.

**Figure 5:**
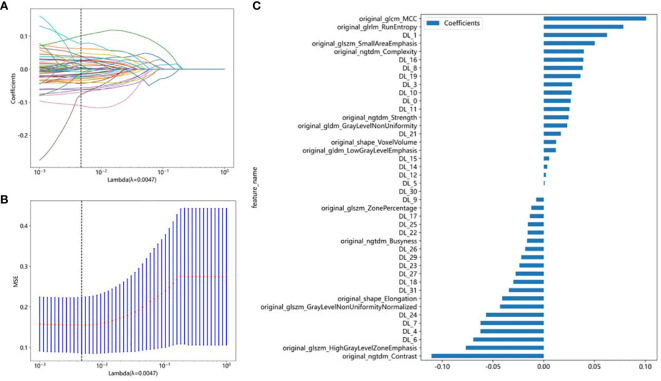
Radiomics combined with DTL feature election using the least absolute shrinkage and selection operator (LASSO) logistic regression model in the test cohort. **(A)** Coefficients of 10-fold cross-validation based on LASSO algorithm. **(B)** MSE of 10-fold cross-validation based on LASSO algorithm. **(C)** Histogram depicting the values of coefficients in the final selected non-zero features.

In this study, a total of 107 handcrafted radiomics features were extracted, including 75 texture features (GLCM: 24, GLDM: 14, GLSZM: 16, GLRLM: 16, NGTDM: 5), 14 shape features, and 18 first-order features. The pre-trained DTL networks extracted 2,048 DTL features and compressed into 32 features after PCA. All features were analyzed using the Spearman rank correlation test and LASSO regression, and all features with non-zero coefficients were selected to construct classification models. Through a LASSO logistic regression model, 23 radiomics features, 32 DTL features, and 42 features obtained by combining radiomics features and DTL features through a fusion method with non-zero coefficients were selected for the establishment of the models. Details of feature extraction and selection of radiomics features, DTL features, and radiomics combined with DTL features can be found in **Figures 3**–**5**.

**Figure 6:**
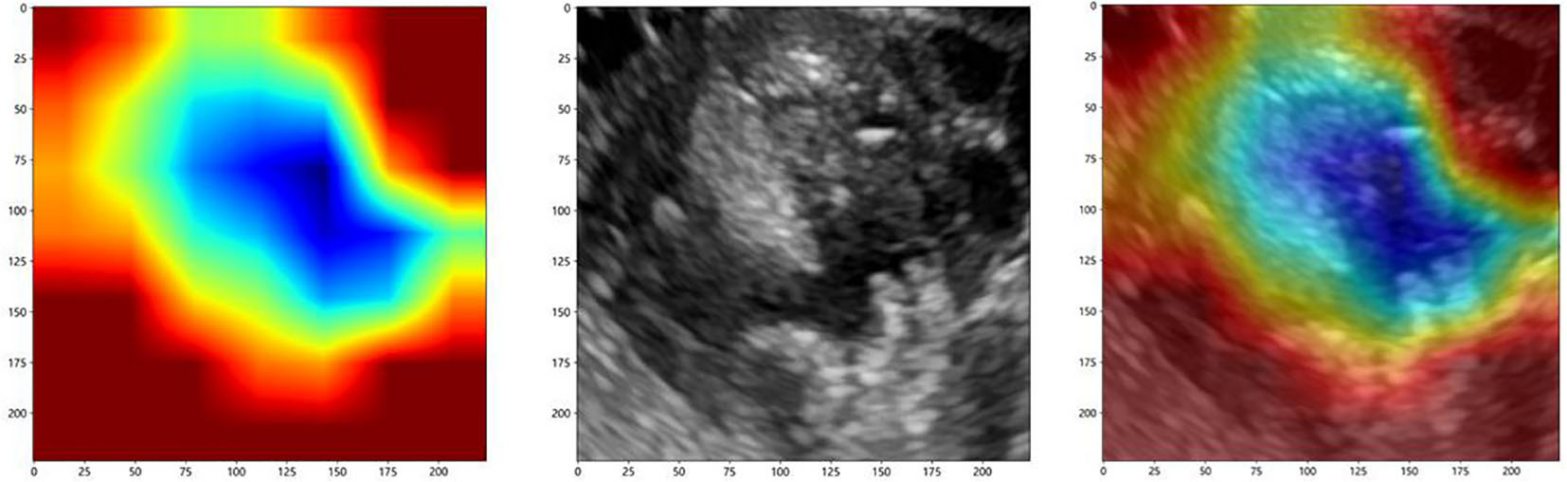
The Grad-CAM visualization for an ovarian mass.

To investigate the interpretability of the DTL features, we visualized the network using the gradient-weighted class activation mapping (Grad-CAM), which could provide a rough localization map to highlight the importance of the ROI for the classification of ovarian masses. The most important regions were marked in red, and the least important regions were marked in blue. **Figure 6** displays the Grad-CAM visualization for an ovarian mass.

### 3.3 Performance comparison of DTL models

To find the best CNN model for extracting DTL features, we compared the performance of Resnet-50, Resnet-152, Resnet-101, Densenet-121, Densenet-201, and Inception v3. After feature extraction and selection, different machine learning models were constructed. We trained and evaluated these machine learning models based on the selected DTL features. Several models, including LR, SVM, KNN, random forest, XGBoost, LightGBM, MLP, NaiveBayes, and GradientBoosting were constructed and compared to determine the most optimal performing model. The results showed that pre-trained Resnet-101 with SVM model achieved the best performance in both training and test cohorts. The AUC, accuracy, sensitivity, specificity, PPV, NPV, and precision were 0.915 (95% CI: 0.888–0.942), 80.6%, 94.4%, 75.5%, 58.7%, 97.3%, and 58.7% in the test cohort. **Table 2** presents the diagnostic indices of these models in the training and test cohorts, including AUC, accuracy, sensitivity, specificity, PPV, NPV, and precision.

**Table 2:** Diagnostic performance of different DTL models.

Cohort	Model	AUC (95% CI)	Accuracy (%)	Sensitivity (%)	Specificity (%)	PPV (%)	NPV (%)	Precision (%)
Training	Resnet-50	0.895 (0.871–0.919)	82.1	82.3	82.1	70.9	89.7	70.9
Training	Resnet-101	0.982 (0.972–0.992)	94.3	93.7	94.6	90.2	96.6	90.2
Training	Resnet-152	0.907 (0.884–0.929)	81.8	85.2	80.0	69.4	91.1	69.4
Training	Densenet-121	0.907 (0.885–0.929)	81.3	88.6	77.4	67.5	92.7	67.5
Training	Densenet-201	0.975 (0.966–0.984)	91.2	92.8	90.4	83.7	96.0	83.7
Training	Inception_v3	0.944 (0.927–0.961)	86.5	88.6	85.4	76.4	93.4	76.4
Test	Resnet-50	0.904 (0.872–0.936)	82.4	84.1	81.7	62.9	93.3	62.9
Test	Resnet-101	0.915 (0.888–0.942)	80.6	94.4	75.5	58.7	97.3	58.7
Test	Resnet-152	0.885 (0.853–0.917)	81.6	83.2	81.0	61.8	92.9	61.8
Test	Densenet-121	0.885 (0.849–0.921)	80.1	89.7	76.6	58.5	95.3	58.5
Test	Densenet-201	0.905 (0.875–0.934)	83.4	82.2	83.8	65.2	92.7	65.2
Test	Inception_v3	0.893 (0.861–0.924)	82.9	87.9	81.0	63.1	94.8	63.1

### 3.4 Performance comparison of radiomics combined with DTL models

**Figure 7:**
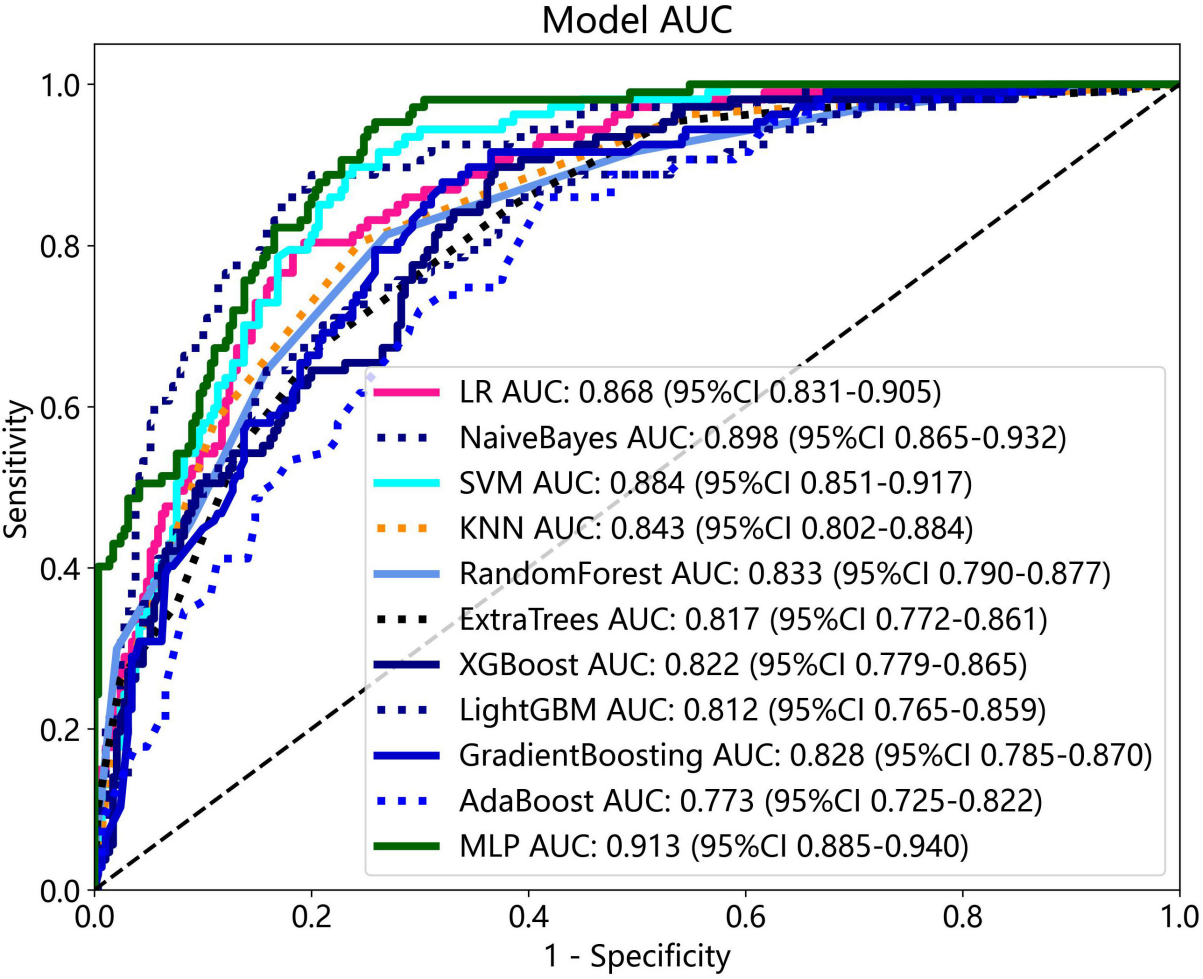
The ROC curves and AUC of different radiomics combined with DTL models in the test cohort.

The radiomics features and DTL features (pre-trained Resnet-101) were fused with concatenation sum strategy to construct different machine learning models, and the MLP model showed the best performance in the test cohort. The AUC, accuracy, sensitivity, specificity, PPV, NPV, and precision were 0.913 (95% CI: 0.885–0.940), 79.6%, 94.4%, 74.1%, 57.4%, 97.3%, and 57.4% respectively. **Table 3** presents the diagnostic indices of these models in the training and test cohort. The ROC curves and AUC values of the different models in the test cohort are shown in **Figure 7**.

**Table 3:** Diagnostic performance of different radiomics combined with DTL models (Resnet-101).

Cohort	Model	AUC (95% CI)	Accuracy (%)	Sensitivity (%)	Specificity (%)	PPV (%)	NPV (%)	Precision (%)
Training	LR	0.947 (0.929–0.964)	89.9	88.2	90.8	83.6	93.5	83.6
Training	SVM	0.989 (0.979–0.999)	96.2	96.6	96.0	92.7	98.2	92.7
Training	KNN	0.974 (0.964–0.983)	91.1	79.7	97.1	93.6	90.0	93.6
Training	Random forest	1.000 (0.999–1.000)	98.8	96.6	100.0	100.0	98.2	100.0
Training	XGBoost	1.000 (0.999–1.000)	99.7	99.2	100.0	100.0	99.6	100.0
Training	LightGBM	0.991 (0.986–0.996)	95.3	97.9	93.9	89.6	98.8	89.6
Training	MLP	0.989 (0.982–0.995)	95.8	94.9	96.2	93.0	97.3	93.0
Training	NaiveBayes	0.898 (0.875–0.922)	81.3	85.7	78.9	68.4	91.2	68.4
Training	GradientBoosting	0.939 (0.921–0.958)	89.2	85.7	91.0	83.5	92.3	83.5
Test	LR	0.868 (0.831–0.905)	80.9	78.5	81.7	61.3	91.2	61.3
Test	SVM	0.884 (0.851–0.917)	79.6	88.8	76.2	57.9	94.8	57.9
Test	KNN	0.843 (0.802–0.884)	80.6	57.9	89.0	66.0	85.1	66.0
Test	Random forest	0.833 (0.790–0.877)	78.8	64.5	84.1	60.0	86.5	60.0
Test	XGBoost	0.822 (0.779–0.865)	70.0	88.8	63.1	47.0	93.8	47.0
Test	LightGBM	0.812 (0.765–0.859)	74.8	73.8	75.2	52.3	88.6	52.3
Test	MLP	0.913 (0.885–0.940)	79.6	94.4	74.1	57.4	97.3	57.4
Test	NaiveBayes	0.898 (0.865–0.932)	82.1	87.9	80.0	61.8	94.7	61.8
Test	GradientBoosting	0.828 (0.785–0.870)	73.0	86.9	67.9	50.0	93.4	50.0

### 3.5 Construction of the nomogram and performance comparison of various feature fusions

All clinical characteristics were used to establish the clinical model due to these characteristics with a p-value <0.05 between the two groups in the training cohort. The clinical features with LR model showed the best performance in the test cohort (AUC: 0.802, 95% CI: 0.756–0.849, accuracy: 69.8%, sensitivity: 85.0%, specificity: 64.1%, PPV: 46.7%, NPV: 92.1%, precision: 46.7%).

**Figure 8:**
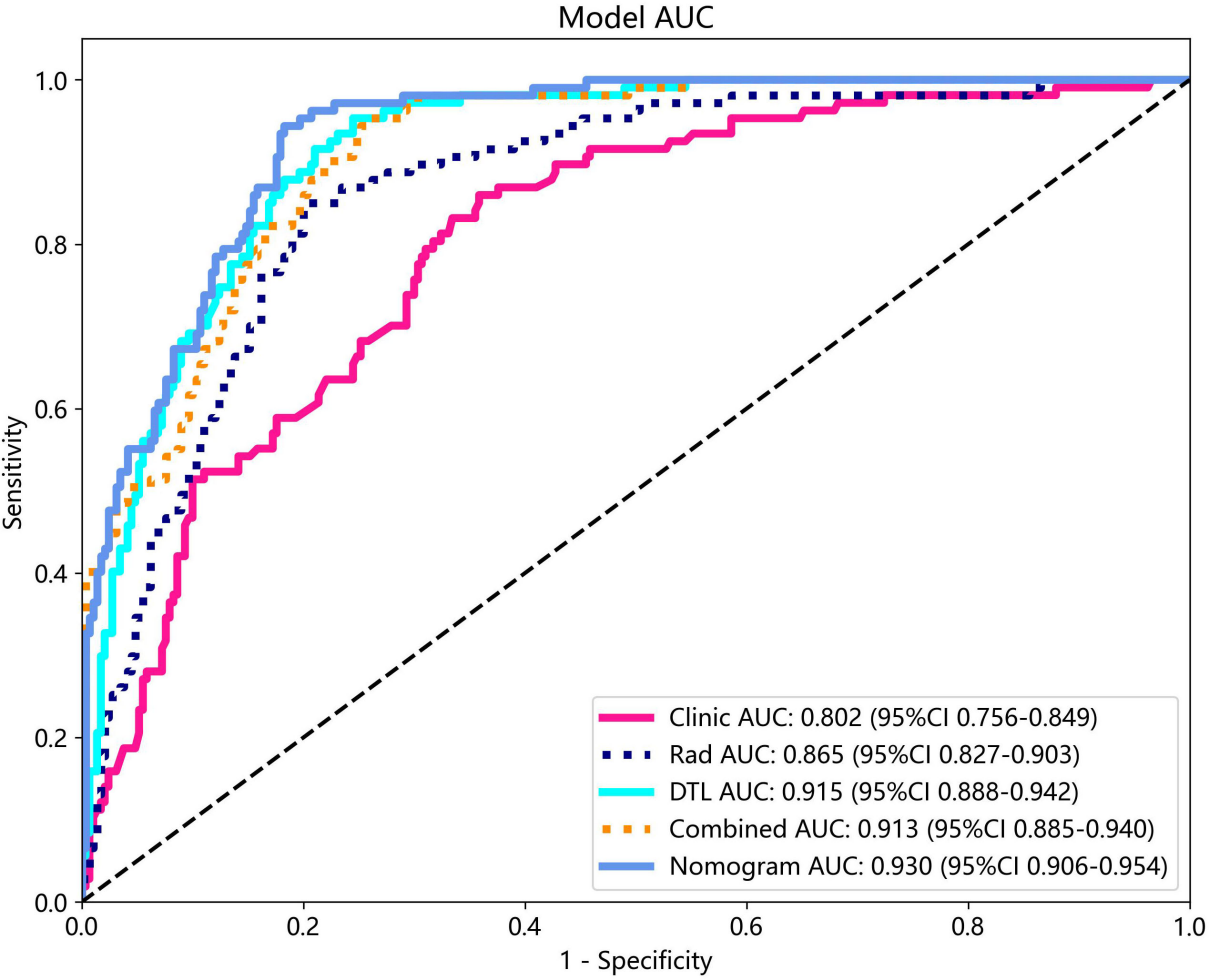
The ROC curves and AUC of clinical, radiomics, DTL, radiomics combined with DTL, and nomogram models in test cohort. Combined refers to radiomics combined with DTL model.

**Figure 9:**
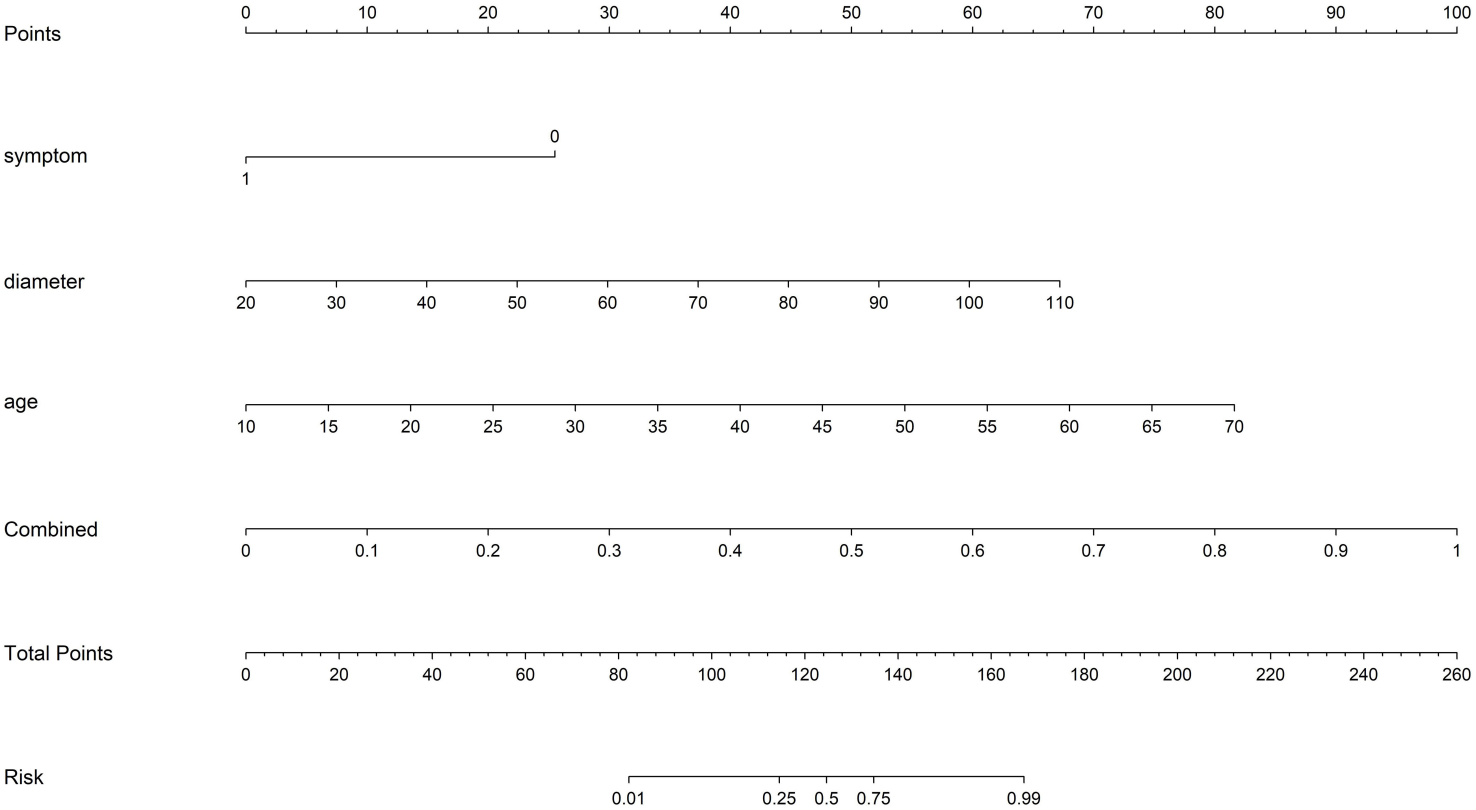
The nomogram with a total score reflecting the probability of malignancy in ovarian masses. Combined refers to radiomics model combined with DTL model.

Various mono-modal features were combined to obtain the optimal subset of fusion features. The nomogram incorporating the clinical features, radiomics features, and DTL features with the LightGBM algorithm demonstrated the highest level of diagnostic performance (AUC: 0.930, 95% CI: 0.906–0.954, accuracy: 84.9%, sensitivity: 93.5%, specificity: 81.7%, PPV: 65.4%, NPV: 97.1%, precision: 65.4%). The diagnostic indices of the clinical, radiomics, and nomogram models in both the training and test cohorts are presented in **Table 4**. **Figure 8** illustrates the ROC curves and AUC values of the different models in the test cohort. **Figure 9** depicts the nomogram for clinical use with a total score reflecting the probability of malignancy in ovarian masses.

**Table 4:** Diagnostic performance of clinical, radiomics, DTL, radiomics combined with DTL, and nomogram models.

Cohort	Model	AUC (95% CI)	Accuracy (%)	Sensitivity (%)	Specificity (%)	PPV (%)	NPV (%)	Precision (%)
Training	Clinical	0.825 (0.794–0.856)	74.2	80.2	71.1	59.6	87.1	59.6
Training	Radiomic	0.924 (0.902–0.947)	86.1	86.1	86.1	76.7	92.1	76.7
Training	DTL	0.982 (0.972–0.992)	94.3	93.7	94.6	90.2	96.6	90.2
Training	Clinical+ Radiomic	0.943 (0.926–0.960)	87.7	89.0	87.0	78.4	93.7	78.4
Training	Radiomic+ DTL	0.989 (0.982–0.995)	95.8	94.9	96.2	93.0	97.3	93.0
Training	Nomogram	0.990 (0.981–0.998)	96.0	95.8	96.2	93.0	97.7	93.0
Test	Clinical	0.802 (0.756–0.849)	69.8	85.0	64.1	46.7	92.1	46.7
Test	Radiomic	0.865 (0.827–0.903)	81.1	84.1	80.0	60.8	93.2	60.8
Test	DTL	0.915 (0.888–0.942)	80.6	94.4	75.5	58.7	97.3	58.7
Test	Clinical+ Radiomic	0.898 (0.866–0.930)	83.6	78.5	85.5	66.7	91.5	66.7
Test	Radiomic+ DTL	0.913 (0.885–0.940)	79.6	94.4	74.1	57.4	97.3	57.4
Test	Nomogram	0.930 (0.906–0.954)	84.9	93.5	81.7	65.4	97.1	65.4

**Figure 10:**
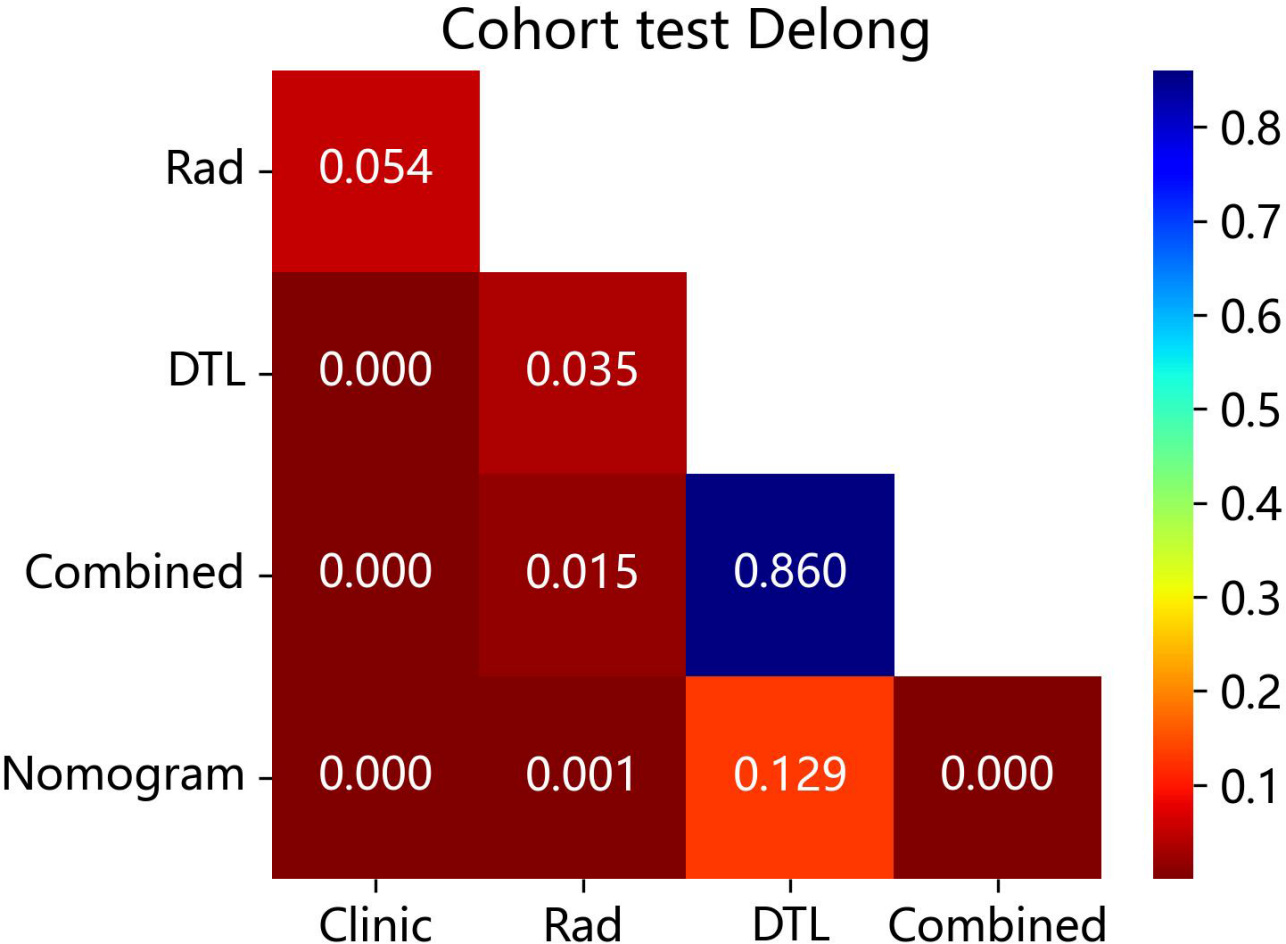
The p-value of the DeLong test between the different models in the test cohort. Combined refers to radiomics model combined with DTL model.

The DeLong test revealed that the AUC comparison between the nomogram and clinical model, between the nomogram and radiomics model, as well as between the nomogram and radiomics model combined with DTL model, was statistically significant (p < 0.05) in the test cohorts, indicating that the nomogram model outperformed these models in the classification of ovarian masses. However, there was no statistical difference in diagnostic performance between the nomogram and DTL model (p = 0.129). **Figure 10** displays the p-value of the DeLong test between different models.

**Figure 11:**
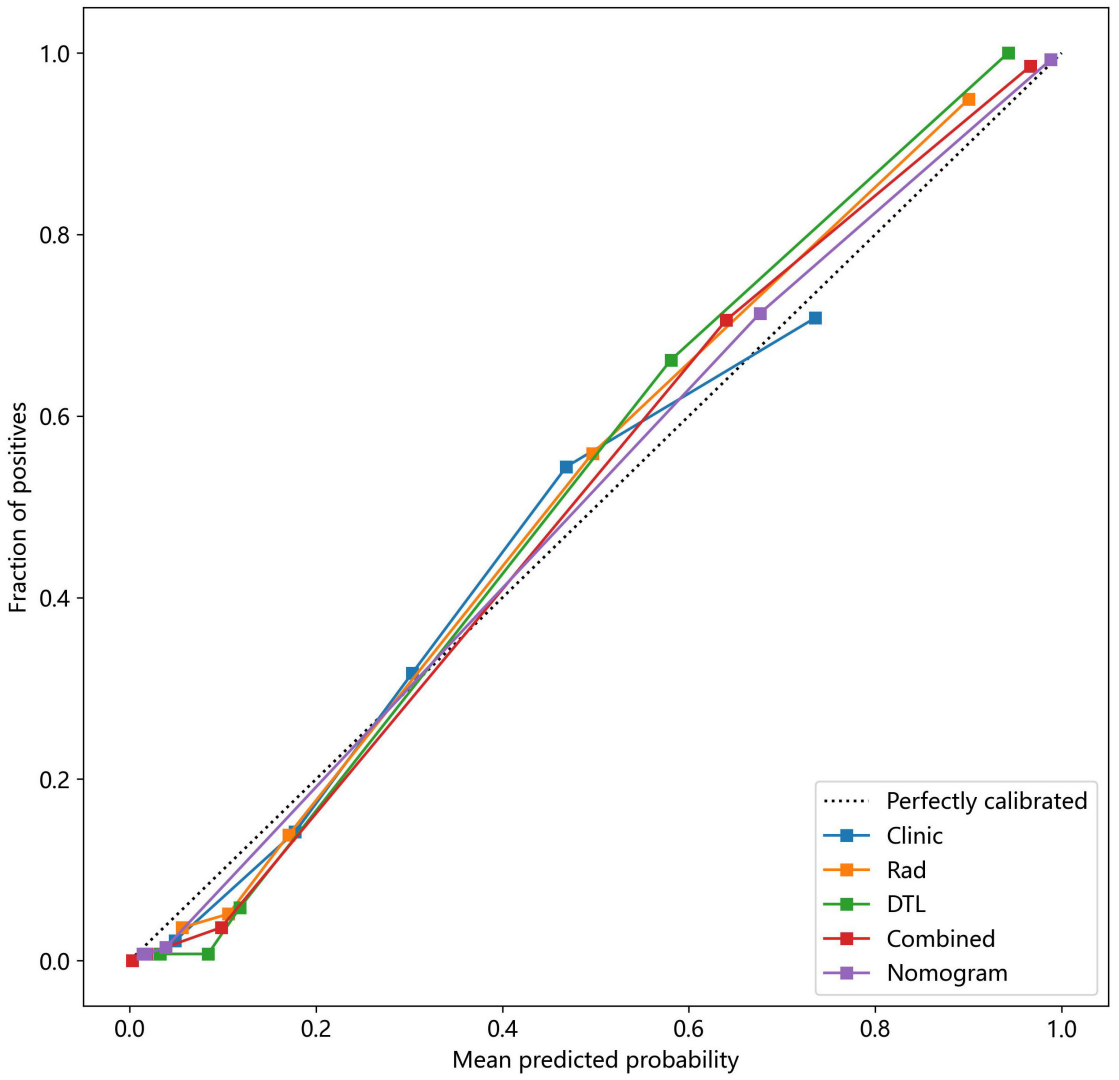
The nomogram calibration curves of the different clinical models in the test cohort. Combined refers to radiomics model combined with DTL model.

The Hosmer–Lemeshow test, used to evaluate the nomogram calibration curves, showed good agreement between the prediction of the nomogram and the actual classification of ovarian masses in the test cohorts (p > 0.05), indicating that the nomogram fitted perfectly. However, the prediction of the rest of the models and the actual classification did not fit very well (p < 0.05). **Table 5** presents the p-value of the Hosmer–Lemeshow test in the test cohorts. **Figure 11** shows the calibration curves in the test cohort.

**Table 5:** p-Value of Hosmer–Lemeshow test.

Cohort	Clinical model	Radiomic model	DTL model	DLR model	Nomogram
Training	<0.001	<0.001	<0.001	<0.001	0.752
Test	<0.001	<0.001	<0.001	<0.001	0.711

**Figure 12:**
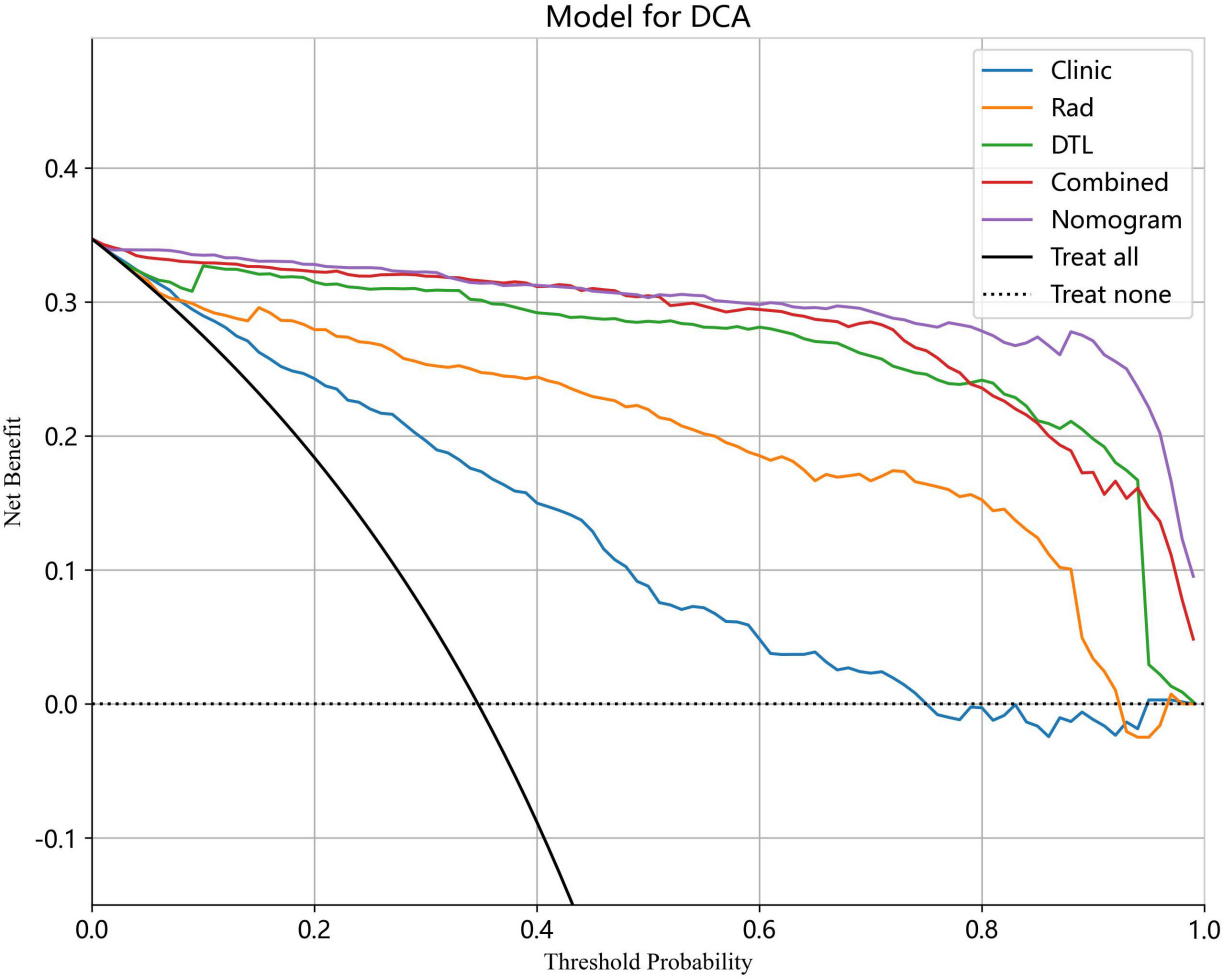
The DCA curves for different models in the test cohort. Combined refers to radiomics model combined with DTL model.

The analysis of the DCA curve demonstrated that, when compared to scenarios without any prediction model, all models significantly improved the intervention outcomes for the patients, and the use of nomogram for automatic classification of ovarian masses has been shown to have the best clinical benefits. **Figure 12** depicts the DCA curves for the different models in the test cohort.

### 3.6 Performance comparison with junior radiologists

**Figure 13:**
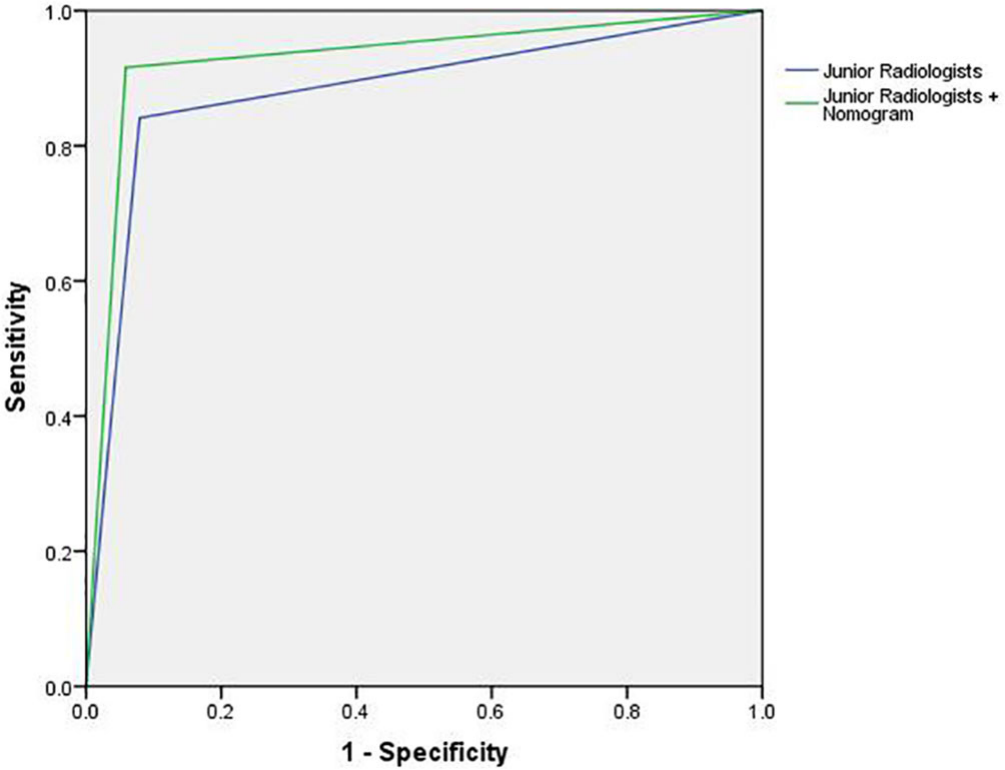
The ROC curves and AUC for junior radiologists with and without the assistance of the nomogram.

Compared to the nomogram, the junior radiologists showed lower diagnostic performance in the test cohort with an average AUC, sensitivity, and specificity of 0.881 (95% CI: 0.857–0.908), 84.1%, and 92.1%, respectively. However, with the assistance of the radiomic nomogram, the junior radiologists exhibited a significant improvement in diagnostic performance, achieving an average AUC, sensitivity, and specificity of 0.929 (95% CI: 0.903–0.950), 91.6%, and 94.1%, respectively. **Figure 13** illustrates the ROC curves and AUC values for junior radiologists with and without the assistance of the nomogram.

## 4 Discussion

O-RADS ultrasound risk stratification and management system enables the stratification of adnexal masses based on morphologic features to indicate the risk of malignancy and offers associated management guidance for each risk category (15). This system has subsequently been validated to have good diagnostic performance for the classification of the lesions in multiple retrospective studies (16–19). Due to the completely different treatment for benign and malignant ovarian diseases, accurate detection of lesions with low risk and intermediate-high risk of malignancy is of great clinical significance. However, as the morphology of ovarian masses in ultrasound image is complex and diverse, and the level of expertise of radiologists varies widely, accurate and rapid discrimination between benign and malignant lesions remains challenging. To solve this problem, in this study, an ultrasound image-based nomogram combining radiomics, DTL, and clinical features was constructed to automatically categorize the ovarian masses into low risk and intermediate-high risk of malignancy lesions. A retrospective study reported that the proportion of malignancy was 0% for O-RADS 2, 3% for O-RADS 3, 35% for O-RADS 4, and 78% for O-RADS 5. Using O-RADS 4 as a threshold achieved a sensitivity of 99% and a specificity of 70% (18). Therefore, ovarian masses with O-RADS 1–3 were considered to be low risk of malignancy lesions, while ovarian masses with O-RADS 4–5 were considered to be intermediate-high risk of malignancy lesions. Following published studies and expert consensus recommending the use of pattern recognition by an experienced radiologist as the most accurate ultrasound method for distinction between benign and malignant ovarian lesions (23–25), we used the judgment of senior radiologists as the standard to evaluate the diagnostic performance of the models.

To find the optimal model, we developed and compared five models, including the clinical model, radiomics model, DTL model, radiomics combined with DTL model, and nomogram combining clinical, radiomics, and DTL features. The results indicated that the nomogram demonstrated better diagnostic performance than the other models for the classification of ovarian masses suggesting that the combination of these three features is particularly advantageous for identifying benign and malignancy lesions. Furthermore, the DeLong test demonstrated that the nomogram model outperformed other models in the classification of ovarian masses with statistical significance (p < 0.05). The nomogram calibration curves showed excellent agreement between the prediction of the nomogram and the actual classification in both the training and test cohorts (p > 0.05). Finally, the results of DCA demonstrated that the use of the nomogram offers significant clinical benefits compared to scenarios without any prediction model.

The nomogram model performed better than the junior radiologists in both the training cohort and test cohort. More importantly, the diagnostic indices for the junior radiologists, such as AUC, sensitivity, and specificity, showed significant improvements with the assistance of the nomogram model, with higher values for AUC (0.881 vs. 0.929), sensitivity (84.1% vs. 91.6%), and specificity (92.1% vs. 94.1%). The result indicated that this nomogram model can enhance the diagnostic performance of junior radiologists, help to supplement medical resources in underdeveloped areas, and provide a new method for rapid ultrasound screening of ovarian cancer.

Due to the retrospective nature of this study, it was difficult to collect sufficient clinical data. As a result, we only collected the patients’ age, maximum lesion diameter, presenting symptoms, menopause status, and presence or absence of ascites. The results showed that there were statistical differences in these clinical characteristics between the two groups (p < 0.05). We found that the mean age of the intermediate-high risk of malignancy group was older than that of the low risk of malignancy group, and the proportion of postmenopausal patients was higher in the intermediate-high risk of malignancy group, which is consistent with the conclusion of previous studies that ovarian cancer affects older women more frequently than younger women (26, 27). Most women with ovarian cancer are asymptomatic or have nonspecific symptoms, such as abdominal pain or distension, at an advanced stage (28, 29). As there were patients with endometriosis in the low risk of malignancy group who usually had symptoms, including dysmenorrhea, chronic pelvic pain, and dyspareunia, the proportion of patients with clinical symptoms was higher in the low risk of malignancy group. However, due to the lack of clinical information, the diagnostic performance of clinical model was not as good as that of the other models.

Radiomics involves the conversion of medical images into mineable high-throughput image features by utilizing sophisticated image-processing techniques enabling the extraction and detection of quantitative data that characterize microscopic tissue aspects beyond the ability of the human eyes (30, 31). These data can be subsequently analyzed using either conventional biostatistics or artificial intelligence methods and correlated with pathology diagnoses based on these processed features (32). Deep learning has also shown remarkable progress in medical image analysis. Resnet (Residual Network) is a type of CNN that avoids the problem of gradient disappearance or explosion by learning residuals resulting in increased network efficiency, accuracy, and execution speed (33). In Resnet-101, the mapping relationship between the original input and output features is gradually learned by adding residual units making the deep neural network learning more stable and efficient. Currently, Resnet-101 is widely used in the field of computer vision, and it achieved the best performance in this study. In recent years, transfer learning, a pre-trained CNN, has gradually been used in various medical image analysis domains because acquiring a large number of medical images is difficult. It can increase model performance in target tasks and minimize overfitting with a small training size by transferring previously learned features from source tasks (34).

Multiple studies have been published regarding the use of machine learning or deep learning models for diagnosis of medical images of ovarian masses (22, 35–37). However, these studies have predominantly focused on discrimination between benign and malignant lesions based on pathology results. The published studies indicated that the artificial intelligence technologies have shown satisfactory predictive ability to diagnose and classify benign and malignant ovarian diseases from medical images (38–40). Furthermore, several studies (28, 41, 42) developed deep learning models to discriminate between borderline and malignant ovarian tumors, and these models have shown promising diagnostic efficiency and provided complementary clinical diagnostic information. As far as we know, no studies have focused on investigating the use of artificial intelligence technology for classification of ovarian masses according to O-RADS.

The following are the limitations of this retrospective study. First, the sample size of the dataset is relatively small, especially for the intermediate-high risk of malignancy group, which may induce potential selection bias. Second, manual segmentation of lesion boundaries may lead to human error potentially omitting image features. Third, this study was a retrospective study, which was prone to sample selection bias. Fourth, the lack of clinical information limited the performance of the clinical model. In the future, larger multicenter prospective trials incorporating a broader range of clinical data are necessary to evaluate the diagnostic performance of the predictive model in clinical practice.

## 5 Conclusions

We first constructed an ultrasound image-based nomogram combining clinical, radiomics, and DTL features to automatically classify the ovarian masses into low risk of malignancy lesions and intermediate-high risk of malignancy lesions according to O-RADS. This model has the potential to improve the level of expertise of junior radiologists as an auxiliary diagnosis tool. Furthermore, this integrated model can provide a fast and effective method for ovarian cancer screening and provide more valuable clinical information for treatment decisions on ovarian masses.

## Data availability statement

The raw data supporting the conclusions of this article will be made available by the authors, without undue reservation.

## Ethics statement

The studies involving humans were approved by the ethical committees of the South China Hospital of Shenzhen University (approval number: HNLS20230112101-A). The studies were conducted in accordance with the local legislation and institutional requirements. Written informed consent for participation was not required from the participants or the participants' legal guardians/next of kin in accordance with the national legislation and institutional requirements.

## Author contributions

LL: Conceptualization, Formal analysis, Investigation, Methodology, Writing – original draft. WC: Data curation, Validation, Writing – review & editing. HT: Data curation, Formal analysis, Validation, Writing – review & editing. BW: Formal analysis, Writing – review & editing. JZ: Investigation, Writing – review & editing. TW: Data curation, Writing – review & editing. YH: Conceptualization, Supervision, Writing – review & editing. GY: Conceptualization, Supervision, Writing – review & editing.
